# Dynamic In Vivo Profiling of DNA Damage and Repair after Radiotherapy Using Canine Patients as a Model

**DOI:** 10.3390/ijms18061176

**Published:** 2017-06-01

**Authors:** Nadine Schulz, Hassan Chaachouay, Katarzyna J. Nytko, Mathias S. Weyland, Malgorzata Roos, Rudolf M. Füchslin, Franco Guscetti, Stephan Scheidegger, Carla Rohrer Bley

**Affiliations:** 1 Division of Radiation Oncology, Vetsuisse Faculty University of Zurich, CH-8057 Zurich, Switzerland; nschulz@vetclinics.uzh.ch (N.S.); Hassan.chaachouay@gmail.com (H.C.); knytko@vetclinics.uzh.ch (K.J.N.); 2 Center for Applied Biotechnology and Molecular Medicine (CABMM), University of Zurich, CH-8057 Zurich, Switzerland; 3 ZHAW School of Engineering, Zurich University of Applied Sciences, CH-8400 Winterthur, Switzerland; weyl@zhaw.ch (M.S.W.); furu@zhaw.ch (R.M.F.); scst@zhaw.ch (S.S.); 4 Department of Biostatistics, Epidemiology Biostatistics and Prevention Institute, Faculty of Medicine, University of Zurich, CH-8001 Zurich, Switzerland; malgorzata.roos@uzh.ch; 5 Institute of Veterinary Pathology, Vetsuisse Faculty University of Zurich, CH-8057 Zurich, Switzerland; franco.guscetti@vetpath.uzh.ch

**Keywords:** DNA damage repair, kinetics, γH2AX-foci, comet assay, dog, radiation, DNA repair model

## Abstract

Time resolved data of DNA damage and repair after radiotherapy elucidates the relation between damage, repair, and cell survival. While well characterized in vitro, little is known about the time-course of DNA damage response in tumors sampled from individual patients. Kinetics of DNA damage after radiotherapy was assessed in eight dogs using repeated in vivo samples of tumor and co-irradiated normal tissue analyzed with comet assay and phosphorylated H2AX (γH2AX) immunohistochemistry. In vivo results were then compared (in silico) with a dynamic mathematical model for DNA damage formation and repair. Maximum %DNA in tail was observed at 15–60 min after irradiation, with a rapid decrease. Time-courses of γH2AX-foci paralleled these findings with a small time delay and were not influenced by covariates. The evolutionary parameter search based on %DNA in tail revealed a good fit of the DNA repair model to in vivo data for pooled sarcoma time-courses, but fits for individual sarcoma time-courses suffer from the heterogeneous nature of the in vivo data. It was possible to follow dynamics of comet tail intensity and γH2AX-foci during a course of radiation using a minimally invasive approach. DNA repair can be quantitatively investigated as time-courses of individual patients by integrating this resulting data into a dynamic mathematical model.

## 1. Introduction

Monitoring the formation of damage in a cell after radiotherapy (RT) and the evaluation of DNA repair markers may be a means to provide information on intrinsic radiosensitivity and radio-responsiveness [1,2,3]. Ionizing radiation used as a cancer treatment relies on the formation of direct DNA damage or on creating sufficient cellular damage leading to double strand breaks (DSBs), which in turn trigger the activation of cellular death pathways. Upon damage, the cells activate a DNA-damage response, consisting of various pathways which will sense the extent of damage and induce an effector pathway either leading to cell death, cell cycle arrest, or DNA damage repair [4]. DNA damage detection from patient samples has been performed for various tumor groups irradiated ex vivo, resulting in robust correlations with radio-responsiveness, as known from clinical behavior [2,5]. Furthermore, monitoring the responses towards damage formation and repair from individual tumors may offer a legitimate chance to monitor cancer treatment and even a possibility to predict clinical response to treatment [6,7]. This is thought to hold true even in spite of the high inter-tumoral and inter-patient heterogeneity that is to be expected from patient samples [6,7]. 

While the time-courses in responses to different DNA damaging agents have been well characterized in cultured tumor cells in vitro, little is known about the time-course of DNA damage response in tumor and normal tissues sampled from individual tumor patients [2,5,6,8,9,10]. Specifically, actual time-courses from patients undergoing radiation therapy are lacking. Time resolved data of DNA damage formation and repair can be used to elucidate the relation between DNA damage formation, repair, and cell survival. It can be expected that, for example, a higher amount of DNA fragments in the tail of a comet assay or residual phosphorylated H2AX foci (γH2AX-foci) are an indication of higher initial radiosensitivity. 

The aim of this study was twofold: first, we wanted to use the minimally invasive sampling techniques (fine needle aspirates (FNAs)) and small biopsies for repeated in vivo patient sampling and subsequent biostatistical quantification of the amounts and time-courses of DNA damage produced by therapeutic ionizing radiation. As a model, sampling was performed in canine tumor tissue as well as co-irradiated normal tissue from patients undergoing treatment. In order to study DNA damage in samples of low cellularity, the comet assay [11,12,13] was used to detect DNA breaks at the level of individual cells in a rapid, sensitive, and simple manner. To validate our data from the alkaline comet assay and to gain additional information, staining of γH2AX-foci was performed in parallel at several time-points after treatment with RT [14]. Second, the clinical in vivo results were then compared (in silico) with a dynamic mathematical model for DNA damage formation and repair. With the postulation of a second order decay (removal) of DNA fragments in the comet tail, the goal of the introduction of clinical patient data into such a tumor-patient model is to identify characteristic kinetic constants and delay times [15]. 

By integrating the repeated, minimally invasive in vivo measurements of clinical patients treated with RT into a mathematical model based data analysis, the potential of a computer simulation of such time-courses was strengthened. This in turn will allow its future use in a more comprehensive framework for the interpretation of obtained patient data and for guidance of therapy. 

## 2. Results

### 2.1. Patient, Tumor, and Radiation Dose Characteristics

Serial samples from eight patients were available. Of the eight dogs, four were female (two spayed) and four were neutered males. The dogs were of various pure (*n* = 5) and mixed breeds (*n* = 3) and a total of five breeds were represented. The ages ranged from 8.17 to 13.67 years with a mean of 11.28 (±1.63) years. The weights ranged from 11.00 to 38.50 kg, with a mean of 23.73 (±8.51) kg. Tumor volumes ranged from 9.24 to 97.91 cm^3^ with a mean of 27.43 cm^3^ (±29.33). Of the eight cases, six tumors (75%) were histologically described as canine soft tissue sarcoma and two (25%) as oral malignant melanoma. Radiation therapy was applied in 5 × 6 Gy over 2.5 weeks in seven patients and in 4 × 8 Gy in one patient (with malignant melanoma).

### 2.2. Comet Assay: Tail Intensities (%DNA in Tail) after Radiotherapy in Tumor Samples

**Figure 1:**
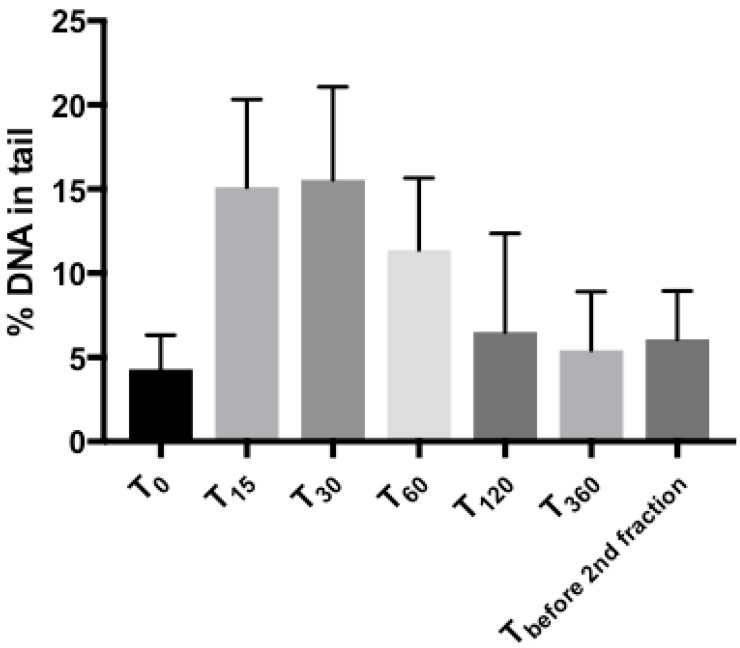
Time-course of DNA damage measured by comet assay. Tail intensity (%DNA in tail) measured at indicated time-points after completion of radiation treatment (T_0_–before treatment, T_15_, T_30_, T_60_, T_120_, T_360_, minutes after treatment, respectively). Mean values ± SD are shown. Significances in differences are displayed in Table 1.

Compared to pre-treatment (T_0_), the measured %DNA tail peaked at 15 to 60 min after RT. The maximum %DNA in tail was observed at 15–60 min after irradiation, with a rapid decrease at 120 min (Figure 1). The median tail intensities differed significantly up to and including the 60 min post-treatment time point (*p* ≤ 0.001). Afterwards, already after 120 and 360 min a rapid decrease was found. The same low intensities were observed before the second fraction, where median tail intensity was not significantly different from T_0_ anymore. (Table 1 and Figure 1).

### 2.3. Phosphorylated H2AX (γH2AX): Number of Positive Cells and Foci per Cell after Radiotherapy in Tumor Samples

**Figure 2:**
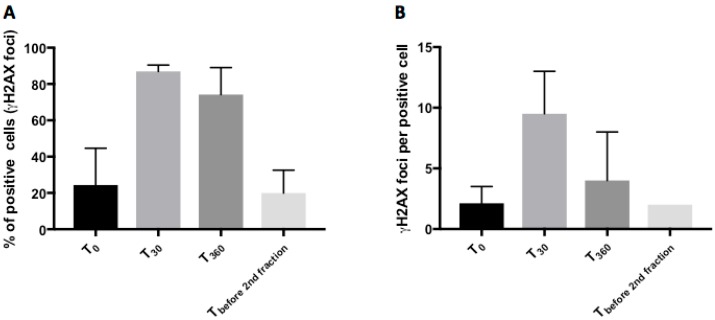
Staining for γH2AX-foci before and after radiation treatment. Number of positive cells (%) (**A**) and number of γH2AX-foci per positive cell (**B**) before and at indicated time-points after completion of the treatment. Mean values ± SD are shown.

**Figure 3:**
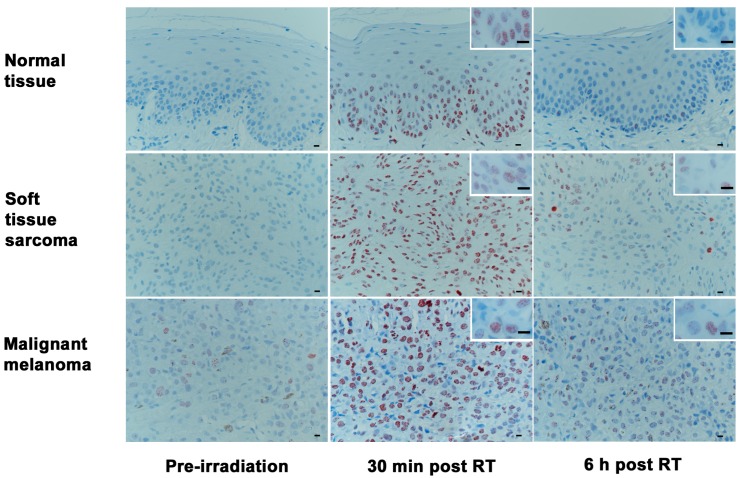
Time-course of γH2AX immunohistochemical labeling in normal epithelium, soft tissue sarcoma, and malignant melanoma. All tissues show low γH2AX reactivity before radiotherapy, high numbers of γH2AX positive cells and foci per cell at 30 min after radiotherapy, and a decrease of the respective numbers at 360 min after radiotherapy. (RT = radiotherapy; bars: overview 20 μm, insert 15 μm); (Immunoperoxidase, haematoxylin-conterstain: brown intranuclear dots represent γH2AX-foci).

Samples from three time-points (T_0_, T_30_, T_360_) of the included patients were analyzed for the number of positive cells (%), as well as foci/cell (Figure 2). Both the amounts of positive cells as well as the numbers of foci per cell at T_30_ and T_360_ were significantly different from those at T_0_ (*p* < 0.001 for positive cells; *p* = 0.002 for foci/cell). As shown in Table 2, both values had returned to baseline before the second fraction. The concurrently sampled co-irradiated normal tissue from the tumor patients followed the same pattern regarding the disappearance of foci (Figure 3).

### 2.4. Covariate Analysis

Due to the time dependence, a linear mixed model approach was applied to detect associations between the median tail intensity, median positive cells, median foci/cell, and covariates such as age, gender, tumor volume, and cancer type. No influence of the covariates or the different fractions of serial measurement was detected.

### 2.5. Baseline Levels of γH2AX in Tumor Biopsy Samples of Various Histologies

**Figure 4:**
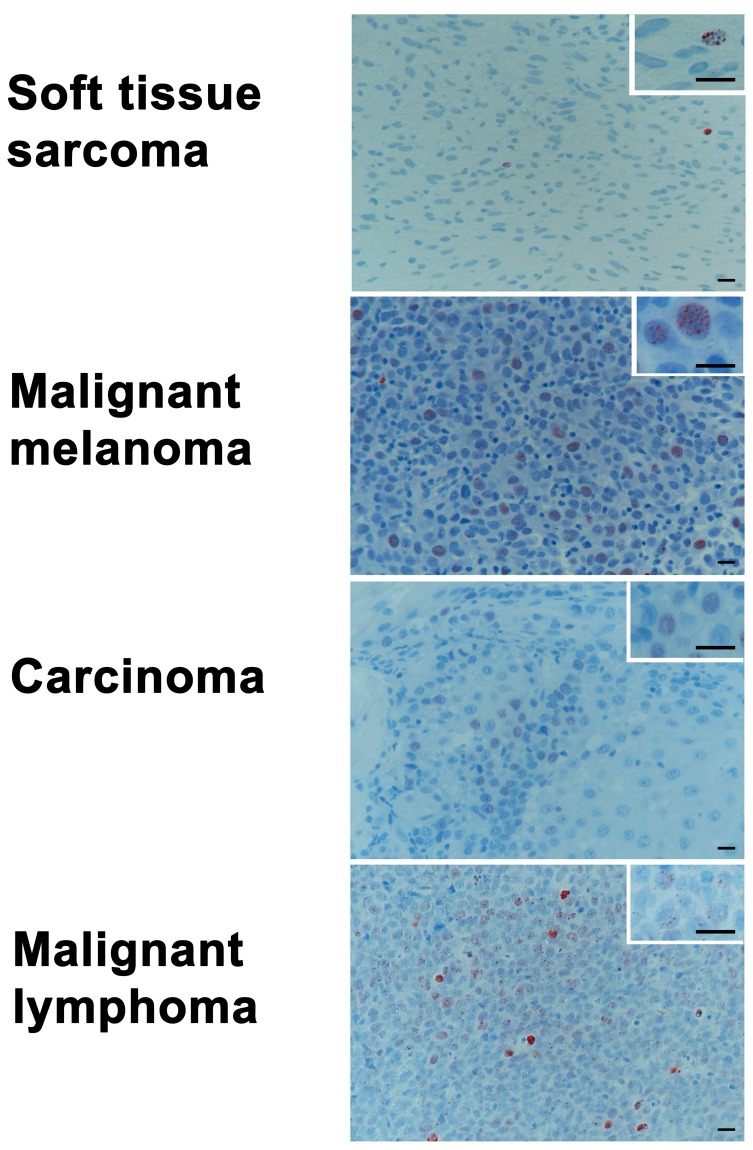
Examples of baseline levels γH2AX immunohistochemical labeling in indicated tumor types. (Bars: overview 20 μm, insert 15 μm); (Immunoperoxidase, haematoxylin-conterstain: brown intranuclear dots represent γH2AX-foci).

Samples of various histologies (soft tissue sarcomas (*n* = 16), malignant melanomas (*n* = 19), carcinomas (*n* = 25), and malignant lymphomas (*n* = 23)) were taken from patients undergoing surgical tumor removal for diagnostic or treatment purposes at the clinic (see Table 3 for patient-specific details). While the sampling groups did not vary in gender (*p* = 0.186), age (*p* = 0.014, malignant lymphoma younger than malignant melanoma patients) and weight (*p* = 0.016, soft tissue sarcomas heavier than malignant melanoma and carcinomas patients) showed some (clinically non-relevant) differences. Histologically, the malignant lymphoma tumor group had significantly higher values in foci/cell (*p* < 0.001) compared to the population with soft tissue sarcoma, malignant melanoma, or carcinomas (Figure 4). In terms of positive cells (*p* = 0.014), the malignant lymphoma tumor group had significantly higher values than the population with soft tissue sarcoma.

### 2.6. Quantification of the Time-Course of DNA Damage and Repair Using Novel Mathematical Modeling

**Figure 5:**
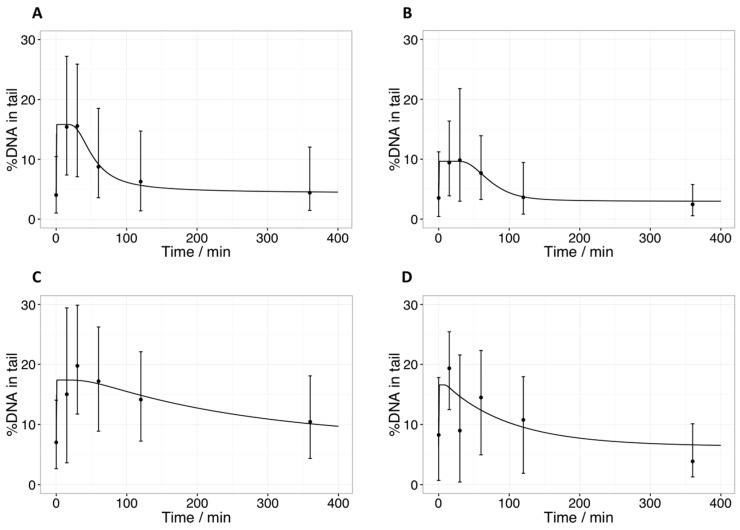
Time resolved comet data fitted by the bio-mathematical model. (**A**) Average of median %DNA in tail of all patients over three fractions for sarcoma cells, with only the fast repair pathway activated; (**B**) median %DNA in tail for the first patient, fraction 1; (**C**) median %DNA in tail for the second patient, fraction 1; (**D**) median %DNA in tail for the second patient, fraction 2. Error bars indicate upper and lower quartiles. The line represents the model prediction with parameters from Table 4.

The evolutionary parameter search (based on the median %DNA in tail) revealed a good fit to the in vivo data for the pooled sarcoma group time-course when both pathways (fast and slow) were active. In order to simplify the model, we tested the case where only the fast pathway was active. This fit had a larger error (Table 4) but in relation to the variability of the data (Figure 5A), it covered the experimental results reasonably well. Selected fits for time-courses of specific fractions are shown in Figure 5B–D. This more detailed display shows how pooling individual fractions and time-courses obtained from different patients can cover up a part of the heterogeneity of the in vivo data. As a consequence, the errors are generally larger than in pooled datasets. The data of the melanoma cases was deemed not suitable for similar analyses, as only time-courses of two patients irradiated at different fractionation schedules were available.

## 3. Discussion

Neither DNA damage induction, nor repair time-courses have been described from patients undergoing radiation therapy up to the present date. Hence, very little is known about the amount of actual DNA damage and the kinetics of repair in tumors in vivo, or in normal tissues under antineoplastic treatment. Herein, we used repeated samples from dogs with primary tumors undergoing therapeutic radiation therapy. Spontaneous tumors in companion animals like dogs are described to offer a unique opportunity as a model for human cancer biology and translational clinical research [15,16]. In contrast to most murine tumor xenograft studies, cancers in dogs arise over the background of an intact immune system and present many features like histological appearance, tumor genetics, molecular targets, biological behavior, and response to conventional therapies, as well as inter-patient tumoral heterogeneity common to humans [15]. Moreover, in many ways, a canine model serves even better than a murine one to study DNA damage repair (DDR) and its defects in vivo, as in rodents, certain repair pathways seem to be less active in comparison to the human mechanisms, displaying a potentially different emphasis and hierarchy of DNA repair pathways [17]. 

The intrinsic radiosensitivity of the tumor cells is a major determinant of the treatment response and outcome, and correlates with a patient’s prognosis [18]. As summarized by McKenna et al., the comet assay technique has also been used in a wide range of human tumor cell lines as well as tissue biopsies and has shown predictive information value of the individual’s sensitivity towards DNA damaging agents [13]. In the herein presented study, the kinetic median tail intensity (%DNA in tail) showed a fast time-course, with no significant remaining differences in tail intensity after 120 min post-radiation in either soft tissue sarcoma or malignant melanoma samples. The alkaline comet assay was chosen because it is capable of detecting and quantifying effects of low, yet clinically relevant radiation doses of 0–6 Gy. Under alkaline conditions, DNA is denatured and both single strand breaks as well as double strand breaks are measured in the tail. In samples of human bladder cancer tissue irradiated ex vivo, evaluation for radiation induced DNA damage levels were measured by this method and the extent of comet formation was found to correlate with cell killing [6]. Furthermore, in this bladder cancer patient cohort, reduced DNA damage sensitivity was associated with poorer treatment outcomes, implying a clinical predictive potential for treatment-induced DNA damage detection with the alkaline comet assay in certain tumors [6,19,20]. However, the alkaline comet assays are able to detect both double and single strand breaks [21,22]. Hence, this assay also detects damage that is not necessarily directly responsible for cell death, as lethality is most frequently observed after non- or mis-repaired DSB. The neutral comet assay, on the other hand, can be modified to detect double strand breaks only, but this requires doses of radiation far higher than the clinically relevant ones. As a general advantage, the comet assay is a relatively inexpensive, simple, and fast technique that can be carried out on single-cell suspensions, and requires only a small number of cells [13]. This is in contrast to the more widely used clonogenic cell survival assay, which measures a surviving fraction of tumor cells after a given clinically relevant dose, but takes a number of weeks to be completed, for example, to obtain the results of clonogenicity of the tumor cells [23]. Showing a strong correlation between the methods of clonogenic survival and alkaline comet assay at clinically relevant doses in tumor cell lines, these measurements of initial as well as residual DNA damage can probably be used to predict radiation sensitivity in patient derived tumor cells as well [19,20,24].

Prior work—including in vivo mouse assays with various cell lines of head and neck carcinoma, as well as in ex vivo assays using different tumor types from patients—has documented radio-responsiveness in sensitive and resistant tumor types to be represented by residual γH2AX-foci [1,2,5]. After antineoplastic treatment, the initiation of DNA repair by the two major responsible pathways is triggered by the phosphorylation of the histone protein H2AX, which leads to formation of γH2AX-molecules at the site of the DSB [4,25,26]. Upon repair, the γH2AX-foci will disappear over a time-course, mostly in concert with DSB rejoining, but it is also suggested that a part of foci removal depends on subsequent steps of DSB rejoining [25,27]. Several reports describe the induction and decline of γH2AX foci in vitro [28,29,30,31], and also in cancer and normal tissues irradiated in/ex vivo [2,5,32,33]. It has been reported that γH2AX foci number in bone marrow cells of mice irradiated with 4 Gy peaked at 1 h after treatment and decreased to baseline already after 4 h, while, in contrast, γH2AX foci were still present in irradiated spermatocytes and round spermatid after 48 h [33]. The kinetics of foci induction and disappearance depends on the dose of ionizing radiation, intrinsic cellular radiosensitivity, cell cycle phase and DNA content, the baseline levels of γH2AX, and, furthermore, on microenvironmental conditions such as tissue oxygen concentration [34]. In accordance with our observations, there is a general pattern of acute increase right after irradiation (5–30 min) with decrease between 0.5 h and 24 h after irradiation, depending on the type of tissue or cells [35]. As a common finding in cultured cancer cells as well as in malignant human tumors of different origins, untreated canine cancers also contain elevated levels of spontaneous γH2AX-foci, which are thought to represent inherent genomic instability [30]. The overall median number of 2.5 foci per cell found herein, ranging from 1.5–2.5 in soft tissue sarcomas to 2.9–4 in malignant lymphoma (e.g., high-grade non-Hodgkin’s lymphoma) was within the range of 1–20 foci per cell described in human cancers and various canine cell lines [30,36]. In line with our data, other recent reports show continuous activation of DNA-repair pathways and constitutive expression of γH2AX in human diffuse large B-cell lymphoma [28,37]. 

The bio-mathematical model chosen can be used to describe the comet assay derived data for sarcoma, even when only one pathway is assumed to be active. The evolutionary parameter search seems to be very sensitive to the constraints set for the optimization runs. In contrast to other models [38,39], the model used in this work summarizes the different steps in a repair pathway by a delayed process. The consequence of this approach is, on one hand, a reduction of parameters but, on the other hand, a model containing Delay Differential Equations (DDE), which makes the evolutionary parameter search more difficult. The interpretation of parameter values derived by fitting the comet data in this work remains challenging at this point, as they vary over several orders of magnitude. The time delays found range from less than one minute (patient 2, fraction 1) to almost 20 min (patient 1, fraction 1), for example. Yet, based on the plots shown in Figure 5 and the large variability in the data, the derived values seem plausible. A better aggregation of parameter values may be reached (1) by modeling distributions instead of relying on median values, and (2) by improving time resolution, especially at the time-point of 240 min. The comparison with time-resolved γH2AX foci signals may be another option; however, the decay of γH2AX foci is slower, possibly due to remaining residues of attached ATM after DNA strands are joined, and therefore could mask the real dynamics of repair (e.g., the time-course of DSB elimination) [40]. In order to optimally distinguish between Homologous Recombination (HR)- and Non-Homologues End-Joining (NHEJ)-pathways, time resolved data from specific markers such as RAD51 for HR, and 53BP1 for NHEJ should be included in the model. To gain deeper insight into the full dynamics of the involved repair processes, dose-rate dependent data could also be included, representing a general limitation of using material from patients with spontaneous tumors. A thorough characterization from in vivo samples is more difficult compared to well-defined cell lines treated in vitro.

In this study, after a first data screening, we decided to average the data of sarcoma patients for the initial modeling. One has to keep in mind that differences in intrinsic radiosensitivity in tumors of the same histological origin exist and can be notable [30,41,42]. It is therefore important not only to investigate the pooled time-course (Figure 5A), but to also review the data before pooling (Figure 5B–D). Thereby, a considerable heterogeneity in the time-courses can be revealed, in particular between fractions of the same patient. While the time-course shown in Figure 5B resembles a scaled version of the pooled time-course, the one shown in Figure 5C is much slower. Furthermore, the median %DNA in tail at T_30_ is higher than the one at T_15_. Since damage is only induced during irradiation and is then assumed to decrease, the model cannot produce such a time-course. Instead, parameter estimates are found that yield a prediction between those two median values. While the variation in medians between T_15_ and T_30_ is well within the range of the error, the discrepancy between the medians and the model prediction lead to an increased error of fit. Figure 5C shows another fraction of the same patient, again presenting a different time-course with a shorter delay and faster decay. The median at T_30_ is low compared to the surrounding medians, resulting in an even larger error of fit. As a limitation of the study, the time-courses from a limited number of patients presented herein do not allow any conclusions to be drawn on individual radioresponsiveness or prognostic relevance of these findings. As a further general limitation of this in vivo approach, minimally invasive and repetitive tumor sampling bears the risk of a mis-representation of the tumor as a whole, due to intra-tumoral heterogeneity in the extent and distribution of malignancy, stromal reaction, inflammatory cells, necrosis, and hypoxia. The sampling heterogeneity with fine needle aspirates and subsequent comet assay might falsely reflect the intrinsic radiosensitivity or repair capacity of a certain area, and manipulation of single tumor cells and tissues can induce further sampling error. Sanguineous contamination may occur, leading to measurement errors with very high DNA intensities in the comet tail, an intensity error that can also be produced by early apoptotic cells [13,43]. Concurrent microscopic evaluations of FNA samples are recommended to assess the representativeness of a sample. These limitations, however, reflect the clinical situation in any predictive testing for individual patients with the goal of personalized medicine and cannot be completely circumvented. However, in patient sample dependent mathematical data modeling, they can be in part attenuated by choosing a high time resolution and large individual sample size. 

At early time-points after RT, quantification of γH2AX foci was impaired due to foci overlap. This is a phenomenon that is also problematic for immunofluorescent staining and computerized quantification. In our evaluation, samples with foci numbers higher than 10 were adjusted as “25 foci” per cell. This was the case in samples taken 30 min and 6 h after irradiation. Furthermore, there is an ongoing debate in the literature regarding what should be considered as foci, which is also related to certain subjectivity in manual scoring [44]. 

In conclusion, we confirm the clinical feasibility of repeated in vivo minimally invasive sampling with FNA as well as small biopsies in order to quantify the amounts and time-courses of DNA damage produced by therapeutic RT. Both dynamics of comet tail intensity and γH2AX-foci could be tracked during a course of radiation and the resulting data could be integrated into a dynamic mathematical model for DNA damage formation and repair. By evaluating not only initial or residual DNA damage, but following a time-course of an individual patient, DNA repair can be quantitatively described.

## 4. Materials and Methods

The current investigation involved samples of canine tumor patients with various types of cancers. In repeated samples from patients treated with RT, DNA damage formation and repair was assessed with comet assay (an assay used to investigate DNA damage in human biomonitoring and genotoxicology) [11,12,13] and γH2AX immunohistochemistry (representing an early event after double-strand break formation) [14,45] at defined time points. The resulting findings were then integrated into the bio-mathematical repair-pathway model in order to perform a model-based analysis of comet data. Additionally, biopsies of various tumors were screened with immunohistochemistry for the amount of pre-treatment γH2AX levels in order to define a baseline of DNA damage in canine tumors.

### 4.1. Patients and Sampling Procedures

Dogs presented to the Division of Radiation Oncology, Vetsuisse Faculty, University of Zurich, Switzerland for treatment of bulky malignant tumors between March 2014 and December 2015 were included in the study. Each dog had a clinical work up (tumor staging) as appropriate to the type of presenting disease. Written owner’s consent was obtained for invasive sampling in this study. This study was carried out in strict accordance with the recommendations and the protocol approved by the Animal Ethics Council of the Canton of Zurich, Switzerland (Permit Numbers: 180/2011, 7 December 2011) and (ZH108/15, 14 January 2016). All invasive sampling procedures were performed under anesthesia, and all efforts were made to minimize suffering. In addition, in order to investigate the basal level of γH2AX, a series of tumor biopsy samples of various histologies (soft tissue sarcomas, malignant melanomas, carcinomas, and lymphomas) were taken from patients undergoing surgical tumor removal for diagnostic or treatment purposes at the clinic. 

For comet assay analysis, tumor cells were collected with a minimally invasive method using fine-needle aspiration (FNA): Sampling was performed with a 5-mL syringe attached to a 22-gauge needle as previously described [46]. The aspirated volume of 10 µL was immediately mixed with a freezing solution of 440 µL fetal calf serum (FCS, Gibco, Thermo Fisher Scientific, Reinach, Switzerland) and 50 µL dimethyl sulfoxide (DMSO, Sigma-Aldrich, Buchs, Switzerland) in pre-cooled cryotubes. Cryotubes were stored at −80 °C until a comet assay was performed. FNA samples from the tumor site were taken in duplicates 15 min before RT and 15, 30, 60, 120 min, and 6 h after RT during the multiple fractions of radiotherapy.

Tumor biopsies for γH2AX immunohistochemistry were collected using 12-gauge Bard^®^ Biopty-Cut^®^ Disposable Core Biopsy Needles, and normal, co-irradiated tissue was sampled with 4 mm punches. Tissue was fixed for 24 h in 10% buffered formalin and embedded into paraffin wax by routine methods. Samples were taken 15 min before, and 30 min and 6 h after RT treatment. 

### 4.2. Treatment

Radiation was delivered with a 6 megavolt (MV) linear accelerator (Clinac iX, Varian, Palo Alto, CA, USA) using either photons or electrons, depending on tumor size and location. Treatment planning was performed on the basis of computer tomography (CT) for photon plans or by hand calculation for electron plans. During treatment, dogs were under general anesthesia using propofol for induction of anesthesia and sevoflurane for maintenance of anesthesia, immobilized in an individually shaped vacuum cushion, and, if required, additionally equipped with a custom-made bite block. The recommendations for specifying dose and volumes as proposed for veterinary medicine were adhered to as proposed in the corresponding literature. The prescribed dose was 30 Gy, delivered in five fractions of 6 Gy applied twice per week, resulting in an overall treatment time of 2.5 weeks [47,48,49]. 

### 4.3. Alkaline Comet Assay

After thawing, the FNA samples were mixed with 9 mL of ice-cold phosphate-buffered saline (PBS) and centrifuged for 10 min (1500 rpm, 4 °C). The pellet was re-suspended in 100 µL of ice-cold PBS. A volume corresponding to approximately 170,000 cells was mixed with 90 µL of agarose (1% low melting point agarose (LMPA, Trevigen, Gaithersburg, MA, USA) in PBS, 37 °C. Subsequently, 25 µL of agarose-cell solution was pipetted onto 20-well slides (Trevigen, Gaithersburg, MA, USA) in duplicate. The loaded slides were then placed at 4 °C for 5 min to allow agarose polymerization, and subsequently placed in lysis buffer for 1 h at 4 °C, followed by incubation twice in ice-cold alkaline electrophoresis solution (pH > 13.0) for 10 min at 4 °C in the dark. After electrophoresis (300 mA, 15–20 min), the slides were incubated twice for 10 min in dH_2_O at room temperature, followed by incubation in 70% ethanol for 5 min at room temperature in the dark. After complete drying, 70 µL of diluted SYBR-Green (1:10,000 in TE, pH 8) was pipetted on every well, and the slides were incubated for 15 min in the dark. The slides were washed twice for 10 min in dH_2_O at room temperature. To provide a quantitative analysis of obtained comets (%DNA in tail, tail intensity), the COMET IV^®^ scoring system was used. 

### 4.4. γH2AX Immunohistochemistry

Information about antibodies, pretreatment, incubation conditions, and visualization are reported in Table 5. For immunohistochemical staining, a Dako Autostainer (Dako, Baar, Switzerland) was used. Three-micrometer (3-µm) sections were mounted on positively charged slides (Superfrost Plus, Thermo Fisher Scientific, Reinach, Switzerland), dried overnight at 37 °C, deparaffinized, rehydrated, and immersed for 10 min in 10% hydrogen peroxide to block endogenous peroxidase activity. Antibody diluent (S2022) and wash buffer (S3006) from Dako (Baar, Switzerland) were used. Negative controls were done omitting the primary antibody.

The quantification of γH2AX positively stained cells and γH2AX foci was performed by manual counting by one investigator (NS) after reaching internal consensus on the procedure. The tumor slides were scanned and analyzed with NanoZoomer 2.0-HT scanscope (Hamamatsu, CH-4500 Solothurn) and visualized using the NDP.view2 software (Hamamatsu). For each tumor, 10 fields of identical size (50× magnification) were set randomly, but dispersed over non-necrotic tumor areas. Furthermore, in each tumor, the number of γH2AX foci were counted in the nuclei of 300 cells.

### 4.5. Bio-Mathematical Modeling

**Figure 6:**
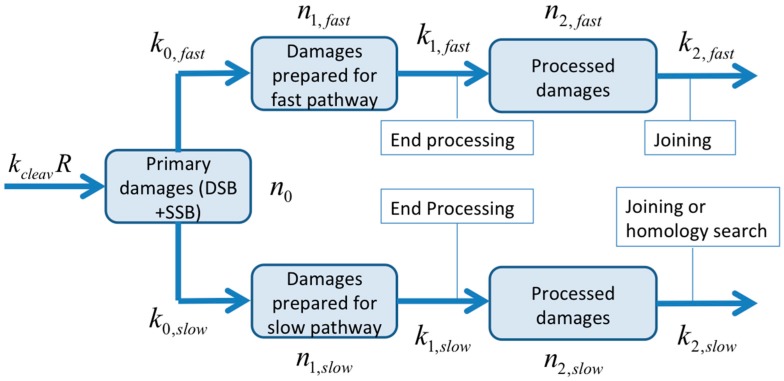
Process flow chart for the two-pathway model. Primary damages ($n_{0}$) are detected by one of the two repair pathways and then transformed to damages prepared for the fast ($n_{1,fast}$) or slow ($n_{1,slow}$) pathway (by a first order kinetics). The damages are further processed and prepared for end-joining, or in the case of HR, homology search. Processed DNA fragments ($n_{2,fast}$ and $n_{2,slow}$) fade away by the final repair step (second order kinetics). The full set of equations of the mathematical model is given in the supplementary materials.

For the bio-mathematical model, the following initial assumption was made: DNA breaks can be processed by (1) a fast repair pathway reflecting Non-Homologues End-Joining (NHEJ) for DSB or SSB-repair by PARP-1/XRCC1/DNA repair module, which may also be involved in the DSB repair by the B-NHEJ backup pathway [50] or by (2) a slower repair pathway (Homologous Recombination (HR) for DSB). Due to the lack of pathway-specific markers in this study, we cannot allocate the two pathways of the model to either HR and/or NHEJ repair mechanisms. We therefore named the two pathways *slow* and *fast*, leaving their connection to HR and NHEJ open. Since SSB is also observable in an alkaline comet assay, it can be expected that DNA-fragments produced by direct DSB will be flanked by those initially produced by SSB. The decision to start with the described two pathways was biologically motivated and based on a preliminary analysis of the comet data from melanoma (data not shown). We found that the two-pathway model did not bear significant advantages over a one-pathway model for the sarcoma data. In particular, the two-pathway model failed to reveal two co-existing repair processes. The parameters resulting from the fitting procedure suggest that one pathway dominates (see Table 4). The model was thus simplified to a one-pathway model by forcing the reaction rates of the slow pathway to 0. Due to the symmetry in the model, this is equivalent to setting the fast pathway to 0. After this simplification, the term *fast* lost its meaning, as only one pathway remains in this model. This entails a modest increase in error of fit due to the loss of four parameters. In Figure 6, a flow chart for the two-pathway model is shown: DNA damages (number $n_{0}$; including SSB and DSB) are produced with an induction rate proportional to the dose rate R ($dn_{0}/dt=k_{cleav}R$; $k_{cleav}$ is a cleavage constant). Since there is a large number of remaining potential target sites (DNA fragments in tail typically below 25%), it is assumed that there is no saturation effect. It has to be pointed out here that this is a simplistic approach for fragment generation. Since a fragment will be cut out of a DNA strand by two hits, one would expect an additional dependence of the absorbed dose *D* at the corresponding time t: $dn_{0}/dt=k_{cleav}R\cdot D$. In addition, pre-existing, non-radiation induced breaks can lead to the liberation of a fragment when a second, radiation-induced break will occur. This process is then, as initially assumed, simply proportional to the dose rate. Both possibilities combined will yield $dn_{0}/dt=k_{cleav}R\cdot \left(\varepsilon +D\right)$ , where ε is a dose-equivalent for pre-existing, not-radiation induced breaks. Simulations with the different models and varying parameter values for ε resulted only in different values for $k_{cleav}$ without significant impact on the terminal course of the number of fragments in the tail. Since the number of data points is limited and experiments were performed at only one dose rate, we decided to use the simplest approach for fragment generation in this study. 

The primary damages ($n_{0}$) can be detected by one of the two repair pathways and then transformed to damages prepared for the fast ($n_{1,fast}$) or the slow ($n_{1,slow}$) pathway. For this process, first order kinetics are assumed with reaction rate constants $k_{0,fast}$ and $k_{0,slow}$, respectively. After detection by a repair-pathway, the damages are processed and prepared for end-joining, or in the case of HR, homology search. This process is also assumed to be of first order with reaction rates $k_{1,fast}$ and $k_{1,slow}$, delayed by $t_{r,fast}$ and $t_{r,slow}$, respectively. The amount (number) of processed damages is denoted by $n_{2,fast}$ and $n_{2,slow}$, respectively. The damages are finally repaired by a second-order process. It is further assumed that all types of damages ($n_{0}$, $n_{1,fast}$, $n_{1,slow}$, $n_{2,fast}$, $n_{2,slow}$) can be transported by electrophoresis and are therefore visible in the comet tail. The total amount of damage predicted by the model is the sum of all damages $n_{i}$ along with a baseline value $b_{n}$ that is co-evolved during optimization. Free DNA fragments fade away by the final repair process step at rates of $k_{2,fast}$ and $k_{2,slow}$, respectively. This process is described by second order kinetics in the model, since two fragments are joined together. The full set of equations of the mathematical model and parameter values is given in the supplementary materials.

For the model-based analysis, the median values of %DNA fragments in comet tail have been calculated. To determine the parameter values in the model, an evolutionary optimization algorithm using a gradient search method was applied [39]. 

### 4.6. Statistical Methods

Data were coded in Excel and analyzed with SPSS version 21. Descriptive statistics such as means, standard deviations, medians, interquartile ranges (IQRs) together with corresponding 95% confidence intervals (95% CIs) were computed. The non-parametric Spearman correlations for associations between two continuous variables were computed. Due to the time dependence, a linear mixed model approach was applied to detect associations between the comet median tail intensity in % (0, 15, 30, 60, 120, 360 min after RT and compared to the beginning of the next fraction), median γH2AX positive stained cells in %, median number of γH2AX foci per cell (0, 30, 360 min after RT and compared to the beginning of the next fraction) adjusted for the following predictors: age, gender, tumor volume, and histological group.

For the analysis of the baseline levels of γH2AX in tumor biopsy samples of various histologies, the Kolmogorov-Smirnov test was applied to check the correctness of normality assumption of the median γH2AX positive stained cells in % and median number of γH2AX foci per cell. The one-way ANOVA together with the Scheffé post-hoc test approach was taken to investigate differences in primary outcomes of median γH2AX positive stained cells in % and median number of γH2AX foci in the nucleus, age, and weight with respect to diagnostic code. The association between the diagnostic code and gender was checked with the Chi^2^-test. Results of statistical analysis with *p*-value < 5% were interpreted as statistically significant.

## Figures and Tables

**Table 1:** Differences of median tail intensity (%DNA in tail), comet assay (%; 95% CI). T_0_, T_15_, T_30_, etc. indicate the time-points in minutes after completion of radiation treatment.

Time-Points	Difference of %DNA in Tail (95% CI)	*p*-Value
T_0_ → T_15_	10.80 (6.56; 15.04)	*p* = 0.001
T_0_ → T_30_	11.24 (6.38; 16.10)	*p* = 0.001
T_0_ → T_60_	7.11 (4.52; 9.71)	*p* < 0.001
T_0_ → T_120_	2.47 (−1.41; 6.36)	*p* = 0.172
T_0_ → T_360_	1.24 (−0.86; 3.33)	*p* = 0.185
T_0_ → T_before 2nd fraction_	1.56 (−0.87; 3.99)	*p* = 0.168

**Table 2:** (**a**) Positive staining for γH2AX-foci (%). T_0_, T_30_, T_360_, etc. indicate the time-points in minutes after completion of radiation treatment. (**b**) Number of γH2AX-foci per positive cell. T_0_, T_30_, T_360_, etc. indicate the time-points in minutes after completion of radiation treatment.

(**a**)		
**Time-Points**	**Median γH2AX Positive Cells (%; 95% CI)**	***p*-Value, Compared to T_0_**
T_0_	20.17 (3.89; 62.36)	
T_30_	87.18 (81.75; 91.78)	*p* < 0.001
T_360_	78.48 (41.88; 85.67)	*p* < 0.001
T_before 2nd fraction_	15.61 (9.80; 41.44)	*p* = 0.764
(**b**)		
**Time-Points**	**Median γH2AX-Foci Per Nucleus (95% CI)**	***p*-Value, Compared to T_0_**
T_0_	2.13 (2.00; 3.50)	
T_30_	8.00 (6.00; 15.00)	*p* < 0.001
T_360_	4.00 (3.00; 8.00)	*p* = 0.002
T_before 2nd fraction_	2.00 (2.00; 2.00)	*p* = 0.163

**Table 3:** Patient specific details for baseline values of γH2AX foci. f = female, fs = female spayed, m = male, mn = male neutered.

Tumor Type	Age (Years) Mean (SD; Range)	Weight (kg) Mean (SD; Range)	Gender	Median Positive Cells (%)	95% CI	*p*-Value	Median Foci/Tumor	95% CI	*p*-Value
Overall (*n* = 83)	9.89 (±2.92; 1.00–15.00)	23.70 (±10.90; 3.60–55.00)	f = 14 fs = 27 m = 19 mn = 23	20.72	21.88; 24.29	0.014	2.50	2.40; 2.90	<0.001
Soft tissue sarcoma (*n* = 16)	9.74 (±1.80; 5.92–12.83	30.40 (±9.88; 14.20–50.70)	f = 3 fs = 7 m = 3 mn = 3	13.41	7.42; 20.59		2.00	1.50; 2.47	
Malignant melanoma (*n* = 19)	11.71 (±2.59; 6.00–15.00)	19.24 (±8.78; 3.60–30.50)	f = 2 fs = 4 m = 7 mn = 6	15.35	12.25; 28.22		2.50	2.14; 2.99	
Carcinoma (*n* = 25)	9.48 (±3.33; 1.00–15.00)	20.79 (±10.23; 4.50–35.50)	f = 6 fs = 6 m = 8 mn = 5	18.12	14.55; 28.67		2.00	2.03; 2.81	
Malignant lymphoma (*n* = 23)	8.94 (±2.67; 3.58–13.75)	25.88 (±11.04; 9.00–55.00)	f = 3 fs = 10 m = 1 mn = 9	31.30	24.72; 34.93		3.00	2.87; 4.04	

**Table 4:** Parameter sets of the bio-mathematical model for the sarcoma group. For the final repair step, second order kinetics was assumed and the data is given in %DNA. The numbers in parentheses in column 1 (sarcoma, pooled) are for a fit of the full model; for the other values in this column, only one pathway is active. The remaining columns contain the fitted parameters of individual patient fractions.

Parameter	Sarcoma, All Patients and Fractions Pooled	Patient 1, Fraction 1	Patient 2, Fraction 1	Patient 2, Fraction 2
$k_{cleav}$/min^−1^	1.93 (1.96)	1.12	1.71	1.71
$k_{0,fast}$/min^−1^	3.93·10^−1^ (1.59 × 10^−1^)	3.76 × 10^−2^	4.35 × 10^−3^	5.24 × 10^−2^
$k_{1,fast}$/min^−1^	2.92·10^−2^ (3.44 × 10^−2^)	2.55 × 10^−2^	5.24 × 10^−2^	1.39 × 10^−1^
$k_{2,fast}$/min^−1^	1.00∙10^−2^ (9.01 × 10^−3^)	8.45 × 10^−2^	2.65 × 10^−2^	1.01
$k_{0,slow}$/min^−1^	- (3.22 × 10^−3^)	-	-	-
$k_{1,slow}$/min^−1^	- (1.01 × 10^−3^)	-	-	-
$k_{2,slow}$/min^−1^	- (1.23 × 10^2^)	-	-	-
$t_{r,slow}$/min	- (7.07)	-	-	-
$t_{r,fast}$/min	1.28·10^1^ (1.09 × 10^1^)	1.93 × 10^1^	8.51 × 10^−1^	3.77
$b_{n}$/% DNA	4.23 (4.09)	2.94	7.12	6.35
Error of fit	1.20 (1.03)	7.31 × 10^−1^	1.23 × 10^1^	6.97 × 10^1^

**Table 5:** Antibodies and incubation conditions. Primary antibody, treatment, and incubation conditions, * HIER = heat − induced epitope retrieval, carried out in a steamer (Pascal S2800, Dako).

Antigen	Vendor	Antibody Type	Catalogue No./Clone	Dilution, Incubation Conditions	Pre-Treatment	Visualization Method	Positive Control
γH2AX Ser 139	Millipore Temecula, CA, USA	mouse mAb, IgG1	05-0636/clone JBW301	1:200, 1.3 h, room temperature	HIER *, 20 min 98 °C, citrate buffer pH 6.0	Envision Kit (Dako)	Irradiated canine tumor tissue

## Supplementary Materials

Supplementary materials can be found at www.mdpi.com/1422-0067/18/6/1176/s1.
