# Supplementary material for: Dynamic In Vivo Profiling of DNA Damage and Repair after Radiotherapy Using Canine Patients as a Model
Source: Int J Mol Sci. 2017 Jun 1;18(6):1176. doi: 10.3390/ijms18061176 (PMC5485999; doi:10.3390/ijms18061176)
Supplement: Supplementary file 1 [file ijms-18-01176-s001.pdf]

Supplementary materials

Bio-mathematical two-pathway model. The initial number of fragments $n_0 = n_0(t)$ is assumed to be produced proportional to the dose rate *R* and a cleavage constant k_{cleav}. The number of fragments $n_{1,fast} = n_{1,fast}(t)$ represents fragments recruited for the fast repair-pathway by a first order kinetics process with the speed constant $k_{0,fast}$. Following the fast repair pathway, the number $n_{2,fast} = n_{2,fast}(t)$ is counting fragments prepared for the fast final repair process by a "delayed" first order kinetics process with the speed constant $k_{1,fast}$, the delay time $t_{r,fast}$. These fragments are removed by a second order process (two fragments are linked together) with the repair constant $k_{2,fast}$. The slow repair pathway has the same structure with the number of fragments recruited for the slow repair pathway $n_{1,slow} = n_{1,slow}(t)$ by a first order kinetics process with the speed constant $k_{0,slow}$ and the number of fragments $n_{2,slow} = n_{2,slow}(t)$ prepared for the slow final repair process by a "delayed" first order kinetics process (speed constant $k_{1,slow}$; delay time $t_{r,slow}$) and removed by second order repair (repair constant $k_{2,slow}$). The total number of free fragments (visible in the Comet tail) is calculated by:

$$n_{Comet} = n_0 + \sum_i (n_{i,fast} + n_{i,slow}) + b_n \qquad (1)$$

where b_n is the base line number of fragments that can be estimated along with the other parameters. All numbers of fragments are scaled to the percentage of DNA in tail (%DNA fragments in tail, corresponding to the experimental data). The induction-, production-, and repair rates are given by the following system of ordinary (ODE) and delay differential equations (DDE):

$$\frac{dn_0}{dt} = k_{cleav}R - (k_{0,fast} + k_{0,slow}) \cdot n_0$$

$$\frac{dn_{1,fast}}{dt} = k_{0,fast}n_0 - k_{1,fast}n_{1,fast}(t - t_{r,fast})$$

$$\frac{dn_{2,fast}}{dt} = k_{1,fast}n_{1,fast}(t - t_{r,fast}) - k_{2,fast}n_{2,fast}^2 \qquad (2)$$

$$\frac{dn_{1,slow}}{dt} = k_{0,slow}n_0 - k_{1,slow}n_{1,slow}(t - t_{r,slow})$$

$$\frac{dn_{2,slow}}{dt} = k_{1,slow}n_{1,slow}(t - t_{r,slow}) - k_{2,slow}n_{2,slow}^2$$

With the initial conditions:

$$n_0(0) = n_{2,fast}(0) = n_{2,slow}(0) = 0$$

and

$$n_{1,fast}(t < 0) = n_{1,slow}(t < 0) = 0$$
